# Disparities in cannabis use and documentation in electronic health records among children and young adults

**DOI:** 10.1038/s41746-023-00885-w

**Published:** 2023-08-08

**Authors:** Nazgol Tavabi, Marium Raza, Mallika Singh, Shahriar Golchin, Harsev Singh, Grant D. Hogue, Ata M. Kiapour

**Affiliations:** 1 Department of Orthopaedic Surgery and Sports Medicine, Boston Children’s Hospital, Boston, MA USA; 2 Harvard Medical School, Boston, MA USA; 3 Department of Computer Science, University of Arizona, Tucson, USA

**Keywords:** Health policy, Population screening, Orthopaedics, Paediatric research

## Abstract

The legalizations of medical and recreational cannabis have generated a great deal of interest in studying the health impacts of cannabis products. Despite increases in cannabis use, its documentation during clinical visits is not yet mainstream. This lack of information hampers efforts to study cannabis’s effects on health outcomes. A clear and in-depth understanding of current trends in cannabis use documentation is necessary to develop proper guidelines to screen and document cannabis use. Here we have developed and used a natural language processing pipeline to evaluate the trends and disparities in cannabis documentation. The pipeline includes a screening step to identify clinical notes with cannabis use documentation which is then fed into a BERT-based classifier to confirm positive use. This pipeline is applied to more than 23 million notes from a large cohort of 370,087 patients seen in a high-volume multi-site pediatric and young adult clinic over a period of 21 years. Our findings show a very low but growing rate of cannabis use documentation (<2%) in electronic health records with significant demographic and socioeconomic disparities in both documentation and positive use, which requires further attention.

## Introduction

In the United States, cannabis is legal for medicinal use in 38 states, and recreational use in 23 states. Up to 22 million Americans 12 years old or older use cannabis annually. This is part of an upwards trend. Daily reported usage of cannabis is increasing, from 2.1% in 2016 to 3.4% in 2019 according to the national survey on drug use. There has also been increased usage among youths^1^. Over 11.8 million young adults report cannabis use. Daily use has increased from 5.9% to 6.9% for 12th graders, 2.9% to 4.4% for 10th graders, and 0.8–1.1% for 8th graders from 2017 to 2020^2^.

Despite this increase in usage, proper documentation of cannabis use has not become mainstream. Such information is vital for a more accurate assessment of cannabis use rates and potential effects on treatment outcomes. For example, cannabis has been shown to influence remodeling of a range of musculoskeletal tissues (e.g., bone)^3^. A recent study on pediatric patients with extremity fractures found significantly increased time to union in those who used cannabis^4^. There is also evidence suggesting cannabis use affects the outcomes of surgeries, including mortality, pain, comorbidities, and revision rates^5,6^. One study found reduced mortality in cannabis users undergoing total hip arthroplasty, total knee arthroplasty, total shoulder arthroplasty, and traumatic femur fixation^7^. Other studies found that cannabis users had higher surgery revision rates^8^ and cannabis users undergoing spine surgery had greater perioperative morbidity^9^.

In terms of pain after surgery, results are contradictory as well. Cannabis users reported lower pain in the operative site in one study of 937 patients^10^, while patients with preoperative cannabis use reported increased pain after major orthopedic surgery in another study of 3793 patients^11^. A different study found that cannabis users had higher total prescribed opioids and longer duration of use^12^. A recent review found that cannabis use in the form of combustive cigarettes represents perioperative risks for induction/anesthesia, post-operative pain, and analgesia in teenagers^13^.

Currently, the most common way of cannabis use documentation in patients’ health records is within unstructured clinical notes, in an unstandardized manner^14^. While studies have proposed the development of standardized screening protocols to streamline documentation^15^, yet no such protocol has been adopted widely. Currently, cannabis itself is referred to in heterogeneous ways within clinical notes, alternatively referred to as MJ, cannabis, weed, CBD, and THC. Some of these terms, specifically weed, and CBD, are nonspecific (i.e., weed also refers to other plants, and CBD also refers to common bile ducts). These terms may also be misspelled. In addition, particularly within pediatric clinical notes, there are mixed mentions of cannabis use among patients and their family members. Additional analysis is needed to distinguish whether the patient is a cannabis user. Overall, the complexity of retrieving cannabis use information from this type of heterogeneous, unstructured data has made it difficult to monitor or study.

One approach to tackle this challenge is the use of natural language processing (NLP) to identify cannabis users. NLP uses linguistic knowledge to extract information (such as cannabis use) from human language by identifying patterns in the data. Applying NLP techniques on clinical notes comes with its own set of challenges such as the presence of noise, heterogeneity, different templates, abbreviations, misspellings, incomplete sentences, etc. Hence, many of the state-of-the-art NLP models do not perform as well when applied directly to clinical notes and the data needs to be thoroughly preprocessed and filtered before being fed into the models. Studies such as Tavabi et al. ^16^, Wang et al. ^17^, and Ling et al. ^18^, developed and evaluated such NLP pipelines and approaches on clinical notes for purposes like cohort identification and building registries. A few studies have focused on identifying substance use from clinical notes using NLP. One study used NLP on clinical notes to identify hospitalized trauma patients with alcohol misuse and demonstrated greater accuracy than EMR-based billing codes^19^. Another study detected alcohol, drug, or nicotine use from unstructured notes using NLP and achieved good performance over a wide breadth of notes^20^. When looking at cannabis use specifically, a separate study developed an NLP algorithm to identify cannabis-related terms, historical mentions, and hypothetical mentions within EHR notes^21^. However, Carrell et al. ^21^ identified 54% of the notes with positive medical cannabis usage automatically and used an NLP-assisted manual review tool to identify the rest, which is a labor and time-intensive process.

In this work, we have developed an NLP pipeline to extract patients’ cannabis use documentation from unstructured electronic health records and among them identify positive cannabis users. We comprehensively assess the changes in cannabis use documentation over the past 21 years along with potential disparities in documentation and positive use in a multi-site high-volume pediatric and young-adults orthopedic and sports medicine (OSM) academic practice, without the need for manual chart review. We have used the data from OSM patients considering the high prevalence of these injuries among children and young adults, and the effects of cannabis use in musculoskeletal tissue healing and remodeling. With a better understanding of how cannabis use is documented, care guidelines for a growing population of cannabis users can improve. In this study, cannabis documentation refers to any mentions of cannabis in the clinical notes, either positive or negative, and cannabis positive use refers to a subset of notes with cannabis documentation in which it is stated that the patient is a cannabis user, whether it’s medical, recreational, lab test, self-reported, etc.

## Results

### Overview of the pipeline

**Fig. 1:**
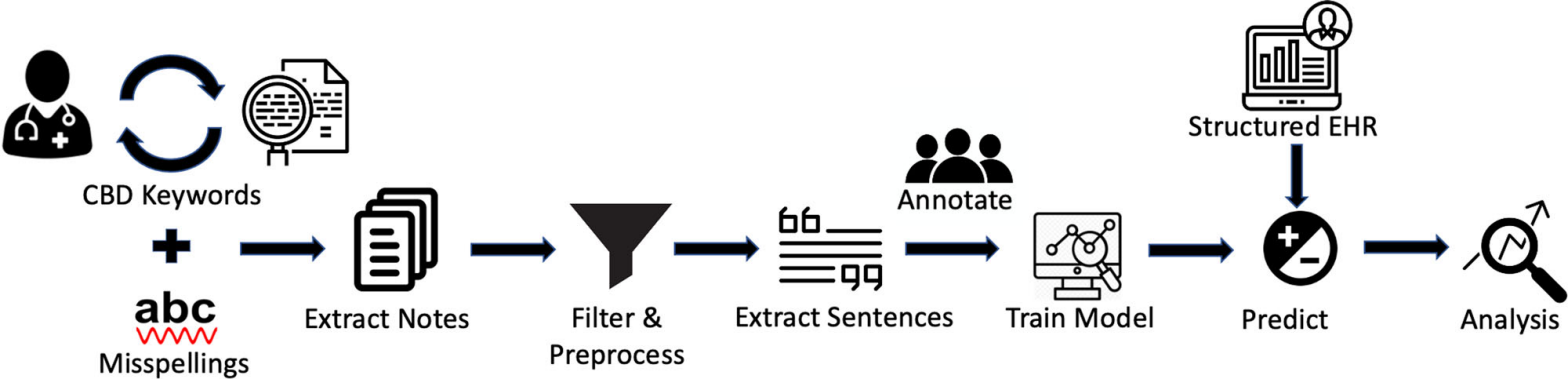
Pipeline overview. Diagram of the pipeline was developed in this study to evaluate cannabis use documentation and to assess disparities in cannabis use among children and young adults with musculoskeletal injuries.

A breakdown of the pipeline developed in this study to evaluate cannabis documentation and positive use in 370,087 unique patients (23,871,108 notes) is shown in Fig. 1. First, through a process of physician-in-the-loop a dictionary of cannabis-related keywords and their possible misspellings was generated. Based on the dictionary, a dataset of clinical notes was extracted, and after preprocessing and filtering out irrelevant notes, the sentences with cannabis-related information were retrieved from the notes. A small percentage of the sentences were then annotated by a group of experts to generate training data for the BERT classifier to confirm positive vs. negative cannabis use. Examples of different types of both positive and negative use are given in Table 1. Afterward, the structured electronic health records (EHR) of the case and control group (patients with predicted positive and negative cannabis usage) such as demographics and a list of diagnoses and procedures were extracted and used for analysis.

**Table 1:** Example notes of positive and negative cannabis use.

Examples of sentences indicating positive cannabis use
Pt admits to smoking marijuana daily
MJ: +
agreed to try marinol with relief, continued scopolamine patch and all other antiemetics changed to prn. Be aware you may be light-headed or dizzy while using marinol RX: DRONABINOL (MARINOL(2.5MG/TAB
Denies drugs or alcohol; states used marijuana "one year ago". Urine tox screen: positive for amphetamines and cannabis.
**Examples of sentences indicating negative cannabis use**
He has not tried any other drugs and denies any use of marijuana, cocaine, heroine, or other drug abuse.
Labs—Urine tox sent—negative for amphetamines, barbiturates, benzos, cannabis, cocaine, opiates, PCP Wrist films and R hand film done—Negative for fracture
I discussed the interactions of stimulant medications with alcohol, which increases the concentration of the stimulant, and marijuana, which increases the heart rate with the use of stimulant medication.
CRAFFT score is 1; he has been in the car when his brother has been smoking marijuana and driving, but his brothers will not allow him to do this.

### Cannabis documentation and positive use prevalence

The baseline demographics of the cohort are presented in Table 2. Of 23,871,108 notes (7.8% OSM notes), 166,530 (2.7% OSM notes) documented cannabis use (23,974 patients), out of which 124,952 notes (2.3% OSM notes) deemed positive use (13,556 patients). The breakdown of patients with positive and negative cannabis use is presented in Table 3. Of all identified positive patients, only 1971 (14.5%) had a medical diagnosis of cannabis use disorder based on either ICD codes or SNOMED CT (Table 6). Meaning if diagnostic codes were used to extract cannabis use information, only 14% of the patients identified using this pipeline would have been retrieved.

**Table 2:** Baseline characteristics of the studied cohort.

Characteristics	Patients
Total sample, no.	370,087
Age (in years) at time of visit, mean (SD)	11.6 (8.27)
*Sex, no (%)*	
Female	194,777 (52.6%)
Male	175,272 (47.3%)
Unknown	38 (<0.1%)
*Race, no. (%)*	
Asian	8461 (2.2%)
Black	17,762 (4.8%)
Hispanic	24,766 (6.7%)
Other	17,933 (4.8%)
White	207,098 (55.9%)
Unavailable	94,040 (25.4%)
SVI, mean (SD)	0.271 (0.244)

The “Other” race group consists of Native American, Alaska native, native Hawaiian or other Pacific Islander, and multi-racial patients.

**Table 3:** Characteristics of patients with and without cannabis documentation.

Characteristics	Patients with documented cannabis use	Patients without documented cannabis use	aOR (95% CI)	*P*-value
Total sample, no.	21,839	348,248	NR	NR
*Sex, no (%)*				
Female	11,471 (52.5%)	183,311(52.6%)	1.074 (1.039, 1.110)	**<0.001**
Male	10,366 (47.5%)	164,901 (47.4%)	NR	NR
Unknown	2 (<0.1%)	36 (<0.1%)	NR	NR
*Race, no. (%)*				
Asian	404 (1.8%)	8057 (2.3%)	0.829 (0.742, 0.926)	**0.001**
Black	3359 (15.4%)	14,403 (4.1%)	3.400 (3.220, 3.590)	**<0.001**
Hispanic	3578 (16.4%)	21,188 (6.1%)	2.245 (2.129, 2.368)	**<0.001**
Other	1237 (5.7%)	16,723 (4.8%)	1.164 (1.001, 1.002)	**<0.001**
White	11,249 (51.5%)	195,849 (56.2%)	NR	NR
Unavailable	2012 (9.2%)	92,028 (26.4%)	NR	NR
SVI, mean (SD)	0.306 (0.265)	0.269 (0.243)	1.001 (1.001, 1.002)	**<0.001**

*P*-values are calculated from generalized linear mixed models. Significant p-values are bold. aOR for female patients is calculated relative to the male patients. aOR for different races is calculated relative to white patients. The “Other” race group consists of Native American, Alaska native, native Hawaiian or other Pacific Islander, and multi-racial patients. For SVI, aOR is calculated per every 0.01 point change.

Bold values identify statistical significance.

**Fig. 2:**
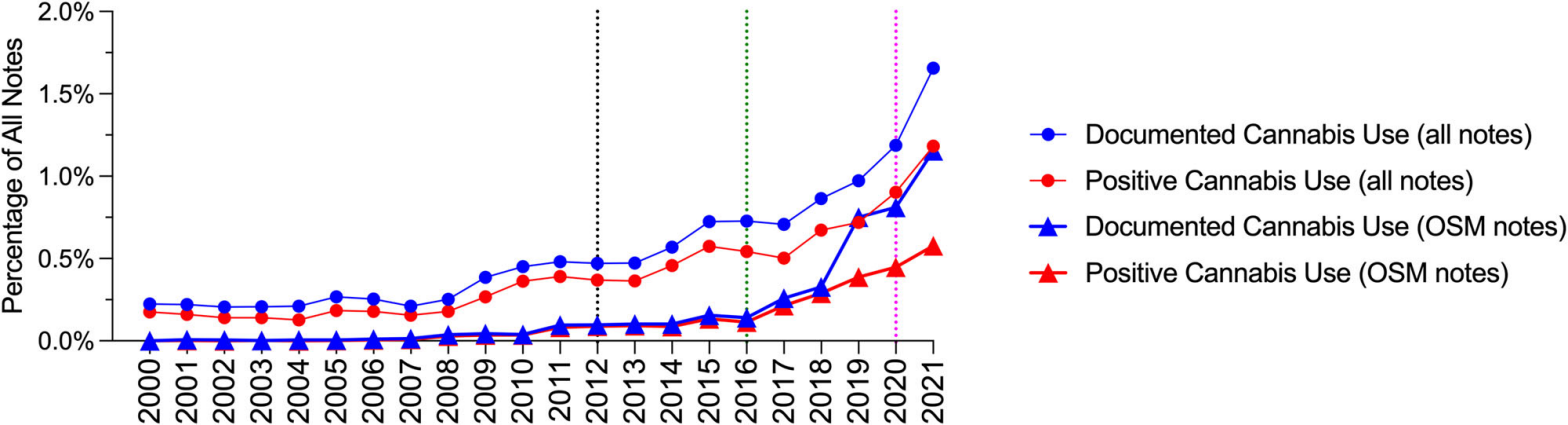
Yearly changes in overall cannabis documentation and use. Cannabis documentation (blue circles) and positive use (red circles) as a percentage of all notes collected each year and cannabis documentation (blue triangles) and positive use (red triangles) in OSM notes as a percentage of all OSM notes collected each year. The vertical dotted lines indicate the legalization of medical (2012, black) and recreational (2016, green) cannabis in Massachusetts, and the declaration of the COVID-19 pandemic (2020, magenta).

From 2000 to 2021, there were increases in both documented and positive cannabis use across all notes (Fig. 2). There were stepwise increases in cannabis documentation across all notes after legalization of medical (2012, black vertical dotted line) and recreational (2016, green vertical dotted line) cannabis (Fig. 2). For the OSM notes, there were increases in documentation and positive cannabis use primarily after 2010 with marked increases after legalization of recreational cannabis (2016, green vertical dotted line; Fig. 2).

**Fig. 3:**
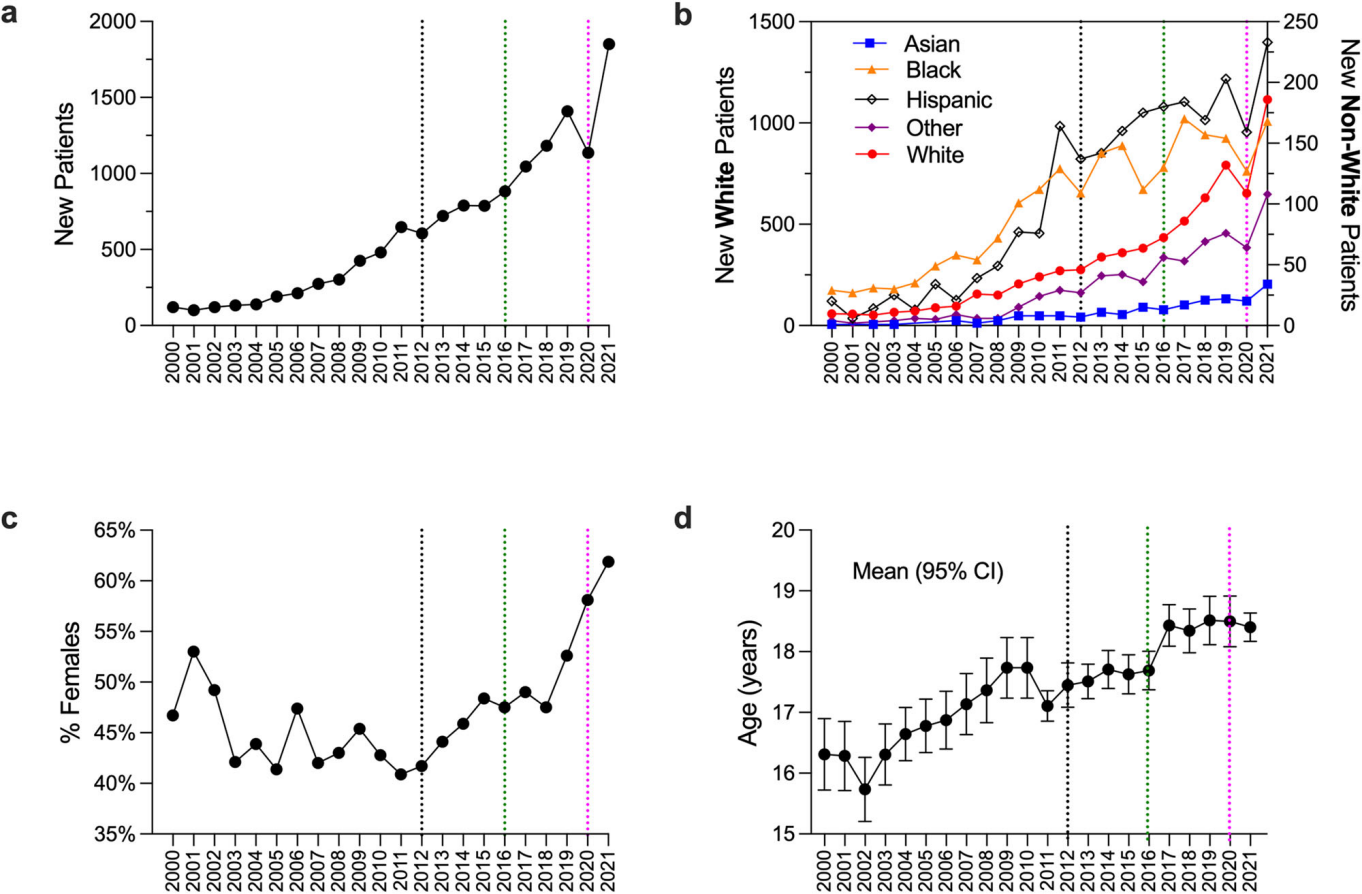
Cannabis rate breakdown in different demographics. **a** Number of newly identified cannabis-positive patients per year. **b** Number of newly identified cannabis-positive patients by each race (other includes Native American, Alaska native, native Hawaiian or other Pacific Islander, and multi-racial). Due to a substantial imbalance in the number of white and non-white patients, white patients are plotted on the left *y*-axis, and non-white patients are plotted on the right *y*-axis. **c** New identified female cannabis-positive patients as a percentage of all new cannabis-positive patients. **d** Average (95% CI) age of newly identified cannabis-positive patients. The vertical dotted lines indicate the legalization of medical (2012, black) and recreational (2016, green) cannabis in Massachusetts, and the declaration of the COVID-19 pandemic (2020, magenta).

From 2000 to 2021, there were increases in the total number of new patients with positive cannabis use (Fig. 3A) with a substantial drop in 2020 corresponding to clinical care restrictions associated with the COVID-19 pandemic (magenta dotted vertical dotted line). Similar trends, with different magnitudes, were observed for new patients with positive cannabis use within each race category (Fig. 3B). While there were no consistent trends in the percentage of new female patients with positive cannabis use prior to 2012 (legalization of medical cannabis), the percentage of new female patients with positive cannabis use increased consistently from 2012 to 2021 (Fig. 3C). Finally, the average age at first recorded positive cannabis use increased from 2000 to 2021 (Fig. 3D).

**Fig. 4:**
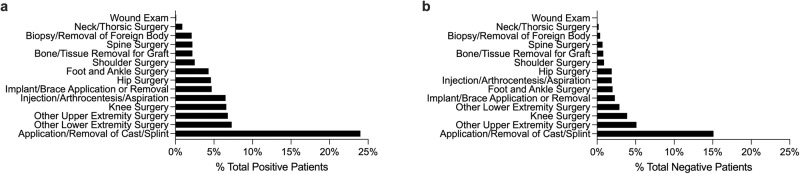
Distribution of musculoskeletal procedures. Distribution of common musculoskeletal procedure groups based on CPT billing codes for patients with positive (**a**) and negative (**b**) cannabis use.

The distribution of musculoskeletal procedures for both positive and negative cohorts was comparable with the application of casting and splints for bone fractures as the most prevalent procedure (Fig. 4).

In the next subsections first, the disparities among patients with and without cannabis documentation are analyzed and later the disparities between patients with and without positive cannabis use are examined.

### Cannabis documentation disparities

Compared to males, females had higher odds of having cannabis use documented in their clinical notes (aOR = 1.074; *P* < 0.001, GLMM test; Table 3). Compared to white patients, Asian patients had lower odds (aOR = 0.829, *P* < 0.001, GLMM test), while black patients (aOR = 3.400, *P* < 0.001, GLMM test), Hispanic patients (aOR = 2.245, *P* < 0.001, GLMM test) and those from other racial backgrounds (aOR = 1.164, *P* < 0.001, GLMM test) had higher odds of cannabis use documentation within clinical notes (Table 3). Patients with higher SVI (Social Vulnerability Index) had higher odds of having cannabis use documented in their clinical notes (aOR = 1.001, *P* < 0.001, GLMM test; Table 3). We observed similar trends when excluding medical cannabis use from the patients with documented cannabis (Supplementary Table 1).

### Cannabis positive use disparities

Compared to males, females had lower odds of positive cannabis use (aOR = 0.957; *P* = 0.038, GLMM test; Table 4). Compared to white patients, Asian patients had lower odds (aOR = 0.651, *P* < 0.001, GLMM test), while black (aOR = 3.222, *P* < 0.001, GLMM test) and Hispanic (aOR = 2.131, *P* < 0.001, GLMM test) patients had higher odds of positive cannabis use (Table 4). Higher SVI was associated with greater odds of positive cannabis use (aOR = 1.002, *P* < 0.001, GLMM test; Table 4).

**Table 4:** Characteristics of positive cannabis use patients compared to negative patients.

Characteristics	Positive patients	Negative patients	aOR (95% CI)	*P*-value
Total sample, no.	13,556	356,531	NR	NR
Age (in years) at the first documented use, mean (SD)	17.9 (5.4)	NR	NR	NR
With diagnosed cannabis abuse disorder, No (%)	1971 (14.5%)	NR	NR	NR
*Sex, no (%)*				
Female	6728 (49.6%)	188,054 (53.7%)	0.957 (0.918, 0.998)	**0.038**
Male	6827 (50.4%)	168,440 (47.2%)	NR	NR
Unknown	1 (<0.1%)	37 (<0.1%)	NR	NR
*Race, no. (%)*				
Asian	206 (1.5%)	8255 (2.3%)	0.651 (0.557, 0.761)	**<0.001**
Black	2144 (15.8%)	15,618 (4.4%)	3.222 (3.014, 3.444)	**<0.001**
Hispanic	2280 (16.8%)	22,486 (6.3%)	2.131 (1.994, 2.278)	**<0.001**
Other	685 (5.1%)	17,275 (4.8%)	1.007 (0.918, 1.104)	0.888
White	7004 (51.7%)	200,094 (56.1%)	NR	NR
Unavailable	1237 (9.1%)	92,803 (26.0%)	NR	NR
SVI, mean (SD)	0.309 (0.266)	0.269 (0.243)	1.002 (1.001, 1.003)	**<0.001**

*P*-values are calculated from generalized linear mixed models. Significant p-values are bold. Patients with diagnosed cannabis abuse disorder are determined based on diagnostic ICD codes. aOR for female patients is calculated relative to the male patients. aOR for different races is calculated relative to white patients. The “Other” race group consists of Native American, Alaska native, native Hawaiian or other Pacific Islander, and multi-racial patients. For SVI, aOR is calculated per every 0.01 point change.

## Discussion

This study demonstrates the utility of NLP in evaluating cannabis documentation and its positive use from heterogeneous and unstructured clinical notes. The generated large-scale database enabled us to investigate the changes in cannabis documentation and positivity rate over the period of 21 years, as well as potential disparities in its documentation and positive use among children and young adults. We observed an increasing trend in cannabis use documentation, in particular over the past decade, with smaller proportions within the OSM clinical notes. These increases included both overall cannabis use documentation (e.g., positive or negative) and positive use. Multiple factors may have contributed to these increasing trends. In particular, the legalization of medical and recreational cannabis use, the development of new cannabis-based medications (e.g., Epidiolex, Marinol) as well as the cultural and policy changes may all have contributed to observed increases in cannabis documentation. Cannabis was legalized for medical use in the state of Massachusetts in late 2012 and for recreational use later in 2016. These legalizations have possibly resulted in increased cannabis usage^22,23^ and/or greater attention paid to the documentation of cannabis use during visits^24,25^. Despite increasing trends, a very small portion of notes overall documented cannabis use (<2%), which for the most part contained insufficient information (i.e., duration, frequencies, amount). These findings are in line with recent studies indicating discrepancies between patient-reported cannabis (e.g., surveys) and cannabis use documentation in health records^26^ while showing a much lower rate than those previously reported.

We saw significant disparities in cannabis documentation and its positive use. Female patients, non-white non-Asian racial groups, and those from a more vulnerable socioeconomic background (i.e., higher SVI) had higher rates of cannabis documentation (whether the patient was asked about cannabis usage or cannabis was discussed with the patient in any way) in their clinical notes. With regards to positive cannabis use, we saw higher rates among male patients, black or Hispanic patients, and those from a more vulnerable socioeconomic background. While the observed discrepancies in positive use are in agreement with those reported in prior studies^27,28^, the demographic and socioeconomic differences in cannabis documentation highlight potential biases and implementation challenges in the proper tracking of cannabis use among children and young adults. The observed racial and socioeconomic disparities in cannabis documentation may also be an underlying factor for the observed higher positive rates among those patients. This highlights the need for a systematic and comprehensive approach to discuss and document cannabis use, regardless of patient demographic and socioeconomic background, which may in turn lead to a more accurate assessment of true disparities in cannabis use.

We saw a comparable distribution of musculoskeletal procedures between positive cannabis users and non-users. This may suggest that the use of cannabis is not primarily influenced by underlying injuries and health conditions. However, whether the use of cannabis has downstream effects on the treatment choices and outcomes requires further evaluation. The high prevalence of cast/splint procedures, often done for treatment of bone fractures, among positive cannabis users along with prior reports of changes in bone remodeling due to cannabis use^3,4,26,29–33^, further highlight the importance of proper cannabis documentation to improve treatment outcomes.

There are several limitations that should be considered when interpreting the current findings. The primary limitation of this study is limited generalizability. This study focused on a single institution, in a single state. Other states may have different policies regulating cannabis access, and patients in those states may have different cultural norms around cannabis use and disclosure. In addition, this study analyzed cannabis usage in a binary (positive/negative) manner since in most cases information on duration, frequency, and dosage were missing. Also, with the exception of toxicology reports and medical cannabis use (e.g., prescription drugs), cannabis use was self-reported which might not be accurate. Such an example could be seen in the fourth case of Table 1, where although the patient denied cannabis use, traces of it was found in their toxicology report. However, it should be mentioned that this is a secondary point in this study with the main takeaway being the low percentage of patients (<7%) being asked about their cannabis use overall. Future studies with confirmatory endpoints (e.g., toxicology reports) are required to assess the true positive rate of cannabis consumption. Despite limitations, this study is among the most comprehensive efforts to evaluate cannabis documentation in a large corpus of clinical notes. Further, to our knowledge, our developed NLP pipeline has the highest performance metrics in capturing a diverse set of cannabis use patterns from clinical notes, which adds to the reliability of the observed findings.

## Methods

This study was performed in line with the principles of the Declaration of Helsinki. Approval was granted by the Institutional Review Board of Boston Children’s Hospital. Considering the retrospective use of available data, the study was exempt from patient consent.

### Data

Following IRB approval, all clinical notes, baseline demographics, and diagnostics and procedures of every patient who has visited the Boston Children’s Hospital OSM clinics (six locations across Massachusetts) between 2000 and 2021 were obtained (370,087 unique patients, 23,871,108 notes). The social vulnerability index (SVI) was calculated based on residence zip code. SVI is a composite index developed by the Centers for Disease Control and Prevention that characterizes community resilience and vulnerability relative to external stressors^34^. It calculates an overall index (0–1), with a higher index indicating greater social vulnerability. For example, an SVI ranking of 0.85 signifies that 85% of zip codes in the nation are less vulnerable than the zip code of interest and 15% of zip codes are more vulnerable. As seen in Table 2 average SVI of Boston Children’s Hospital patients is 0.271. Additionally, current procedural terminology (CPT) billing codes were used to identify the most common musculoskeletal procedures within the study population and diagnosis codes were used to identify patients with cannabis-related diagnoses.

### Natural language processing (NLP) model

An NLP approach was developed to first identify notes containing cannabis-related terms (screening step) and then classify them into positive (endorsed cannabis use) or negative (no cannabis use). For the screening step, first, a cannabis-related dictionary was generated with a physician-in-the-loop approach. Initially, A few seed words (Marijuana, Cannabis, CBD, Weed, THC) and medical terms (Tetrahydrocannabinol, Epidiolex, Cannabidiol, etc.) from FDA-approved cannabis drugs were chosen and notes containing them were collected. Afterward, based on the notes collected, other keywords (such as MJ) were identified and added to the dictionary. The full list of keywords in the dictionary is presented in the supplements.

Additionally, possible misspellings of the keywords were identified and added to the dictionary. In order to add misspellings of dictionary keywords, words from all the notes were gathered and their character *N*-grams (*N* = 1–3) were computed. The cosine similarity of each word’s *N*-gram and *N*-gram of the correct word was computed, and words with higher similarity than a certain threshold were identified. The threshold was chosen manually for keywords in the cannabis-related dictionary. List of keywords identified as misspellings are presented in supplements. Similar approaches have been previously used by other groups to identify and correct misspellings^35–37^. Below is an example of how a misspelled word is identified.

*A* = *N*-grams of Marijuana (correct spelling of a seed word) = [*A*:3, *JU*:1*, AH*:0…]

*B* = *N*-grams of Marijuahana (possible incorrect spelling of a seed word) = [*A*:4, *H*:1, *AH*:1*, AHA*:1…]

Cosine Similarity (*A, B*) = $\frac{{A}\,\cdot \,{B}}{\left|A\right|\left|B\right|}$ = 0.90 > 0.70 (misspelling threshold set for Marijuana)

➔Marijuahana is identified as a misspelling of Marijuana and it is added as another seed word.

**Fig. 5:**
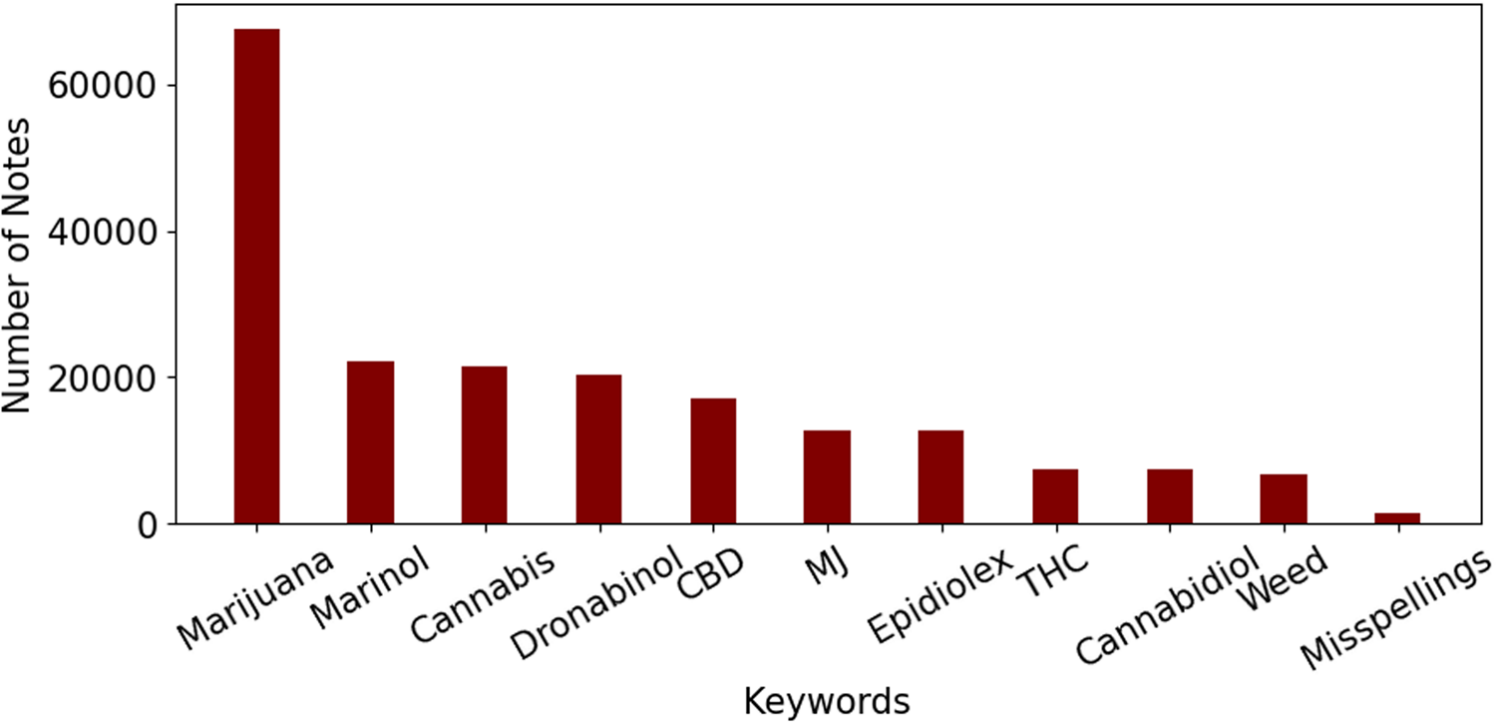
Histogram of keywords used for extracting notes. The keywords used to extract clinical notes and the number of notes containing each of them. Keywords like Syndros and Cesamet which appeared in <10 notes are not included in the graph.

When the dictionary was finalized, all the notes in the dataset (*n* = 23,871,108) were screened for cannabis-related terms. Figure 5 shows the number of notes identified with each keyword (some notes contained more than one keyword).

After finding the notes with any of the keywords in the dictionary, the dataset was first filtered and preprocessed. The first step was to remove the notes that were incorrectly marked as cannabis-related. As mentioned earlier words like CBD or weed can also be used in other contexts. The acronym CBD is also used for common bile duct and is used in notes related to gastrointestinal conditions. Also, the keyword weed is used in notes related to allergy detection. To remove these irrelevant notes, 2 other sets of keywords were compiled. Words like abdominal, gallbladder, pancreas, common bile duct, etc., were used to identify and filter out notes related to the common bile duct, and words like pollen and allergy and their variations were used for allergy-related notes. The full list of keywords for filtering out notes related to the common bile duct and allergy towards weed is given in supplements. Additionally, cannabis-related notes which correspond to very young patients (under 7) were primarily related to the cannabis use in their household and not by the patient, especially during pregnancy. Table 5 shows a few examples for patients under age 7. Since the purpose of this study is to identify patients with direct cannabis use, patients younger than 7 years old were removed from the dataset.

**Table 5:** Examples of cannabis-related notes for patients under 7.

Other tobacco history: Mother smoked but has quit, also use of MJ during pregnancy but has quit as well.
Birth Hx: Exposed to maternal THC in utero.
Pregnancy is complicated by late care, inconsistent prenatal care, marijuana use, and viral illness 5 days prior to delivery.
He lives with his mother and occasionally with his grandmother in the housing projects where she says he is exposed to cigarette and marijuana smoke in the hallways.

After the preprocessing step, the resulting dataset was used to analyze cannabis documentation. Additionally, the same dataset was used in the next steps of the pipeline for identifying positive cannabis use. The types of extracted notes were very heterogeneous, resulting in a collection of different note contents and formats. Some notes had form templates with different fields and incomplete sentences and in some others, the note contained full sentences with a lot of detailed narratives. Since usually only a small section of the note is related to cannabis use, to minimize unwanted bias related to note types (i.e., template and content), only the sentences containing the cannabis-related keywords were extracted and used as input to the classifier.

**Fig. 6:**
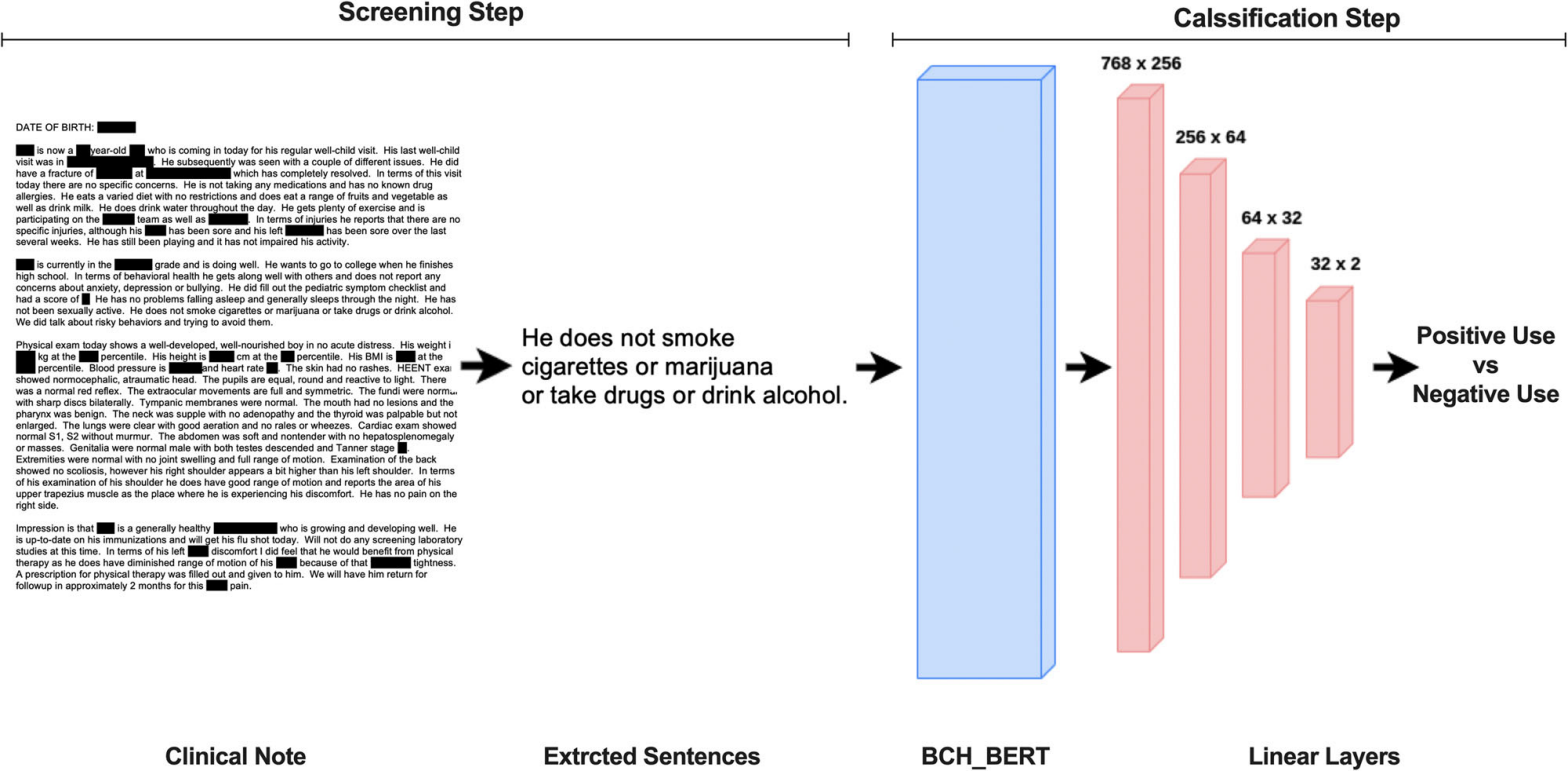
NLP model for cannabis use classification. The deep learning model is used to classify extracted sentences to identify positive cannabis use.

After the screening step, the model bidirectional encoder representations from the transformers (BERT) model were used to classify the extracted sentences. BERT is an NLP model developed and trained by Google, which has been widely used across multiple domains, including healthcare^38^. BERT models provide contextual representations of words and sentences, which can then be used in classification. Domain-specific BERT models yield significant improvement, hence for this project we used ClinicalBERT^39^, which has been pretrained on medical literature (PubMed corpus) and publicly available MIMIC dataset^40^. We further pre trained the ClinicalBERT on all the available notes from Boston Children’s Hospital (BCH BERT; 23,871,108 notes for 1 epoch) so the model would be more familiar with the language used in the clinical notes. We then used the following deep learning model (Fig. 6) to classify the extracted sentences from the screening step into positive use (i.e., self-reported cannabis use by the patient or guardians currently or in the past, positive use reports by the clinical care team or toxicology reports) or negative use (i.e., patient or guardian reported no use, negative use confirmation by clinical care team or toxicology reports, discussions of pros and cons of cannabis use with no direct indication of positive use, reported abuse for someone other than the patient). The model was trained and tested on a diverse set of manually labeled sentences (*n* = 3835, 73% positive, 27% negative, 80% train, 10% validation,10% test). Note that the imbalance towards the positive class is due to the nature of the problem. Since the input to the model is all the notes containing cannabis-related keywords, and inquiring about the cannabis usage of a patient is not currently part of routine clinical care, there is a higher probability of positive cannabis usage when cannabis is mentioned in the notes.

**Fig. 7:**
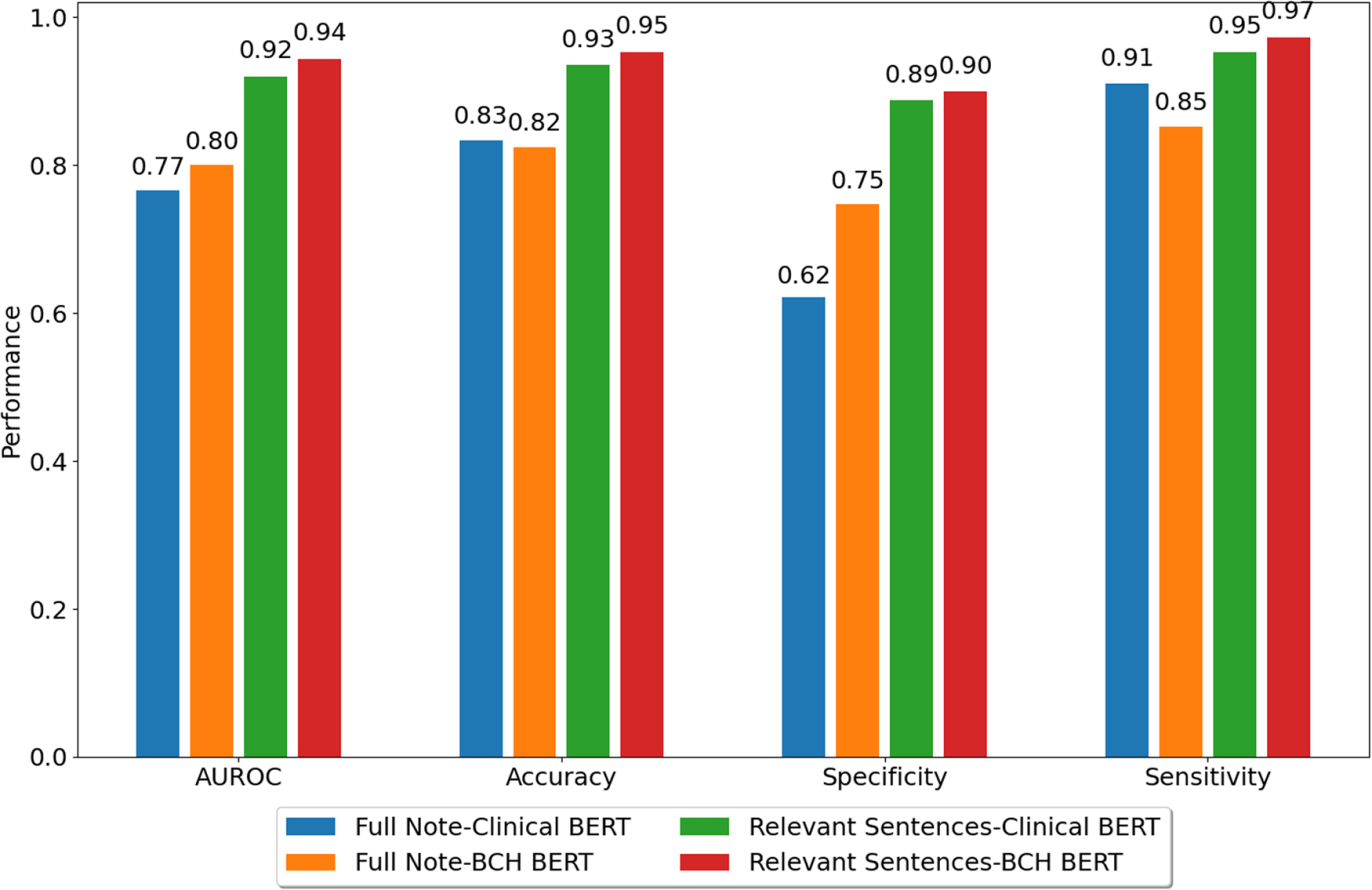
NLP model performance comparisons. Performance comparison of our final model (BCH BERT on relevant sentences) and other models. Comparing, having the full note as the input vs. only the relevant sentences as well as, having ClinicalBERT vs the same model further pretrained on BCH data (BCH BERT).

The training was done for 7 epochs on an NVIDIA Titan RTX GPU. The model achieved an accuracy of 0.95, an area under the ROC curve of 0.94, a sensitivity of 0.97, and a specificity of 0.90 on the test set (Fig. 7). To assess the relative performance of our pipeline compared to alternative strategies, we repeated the classification tasks using ClinicalBERT versus BCH BERT and using the whole note versus selected relevant sentences. Our pipeline outperformed alternative strategies in identifying positive cases of cannabis use (Fig. 7).

After the model was trained on the annotated subset of the data, it was used to classify the entire preprocessed dataset, the cannabis documentation dataset, into positive and negative use.

### Evaluation of disparities in documented cannabis use

Disparities in the data were evaluated in two different settings. First, patients with documented cannabis use vs. patients with no documents mentioning cannabis use. Second, patients with positive cannabis use vs. patients with negative cannabis use. In the first setting the group with no documented cannabis use were never asked about cannabis usage and cannabis was not discussed in any of their notes whereas the other group had mentions of cannabis in at least one note which could be either a positive or a negative use.

A generalized linear mixed model (GLMM) was used to evaluate the associations between sex, race, and SVI in the rate of documented cannabis use (first setting). Adjusted odds ratios (aOR) were calculated and considered significant at *P* < 0.05 (SPSS v27) between those with documented cannabis use (positive or negative) and those with no cannabis use documentation. The aOR was calculated for the female sex compared to the male sex and for Asian, black, Hispanic, and other races compared to the white race. For SVI, the aOR was calculated for every 0.01 point change in SVI. The analysis was repeated after excluding those with medical cannabis use. The same framework was applied to assess disparities in positive vs negative cannabis users with regard to sex, race, and SVI (second setting).

### Diagnostic codes for cannabis misuse

This step was done to identify patients with diagnosed cannabis use which are compared with the patients identified by our NLP pipeline. International Classification of Diseases (ICD) and Systematized Nomenclature of Medicine Clinical Terms (SNOMED CT) are two different coding systems used for diagnosing patients. ICD codes are mostly used after the care is completed for example for billing purposes, whereas SNOMED CT is used directly by healthcare providers during the process of care. There are different ICD codes and SNOMED CTs related to cannabis use. To identify them, we extracted all diagnoses with the keyword “cannabis” in their description, and after they were reviewed and confirmed, all cases with those codes and all the patients diagnosed with them were extracted. Table 6 shows identified diagnostic codes, their descriptions, and their frequency. A patient might get diagnosed with the same code or a similar code more than once.

**Table 6:** Cannabis use diagnostic codes.

Diagnosis code	Source vocabulary	Description	Count
F12	ICD-10-CM	Cannabis-related disorders	10,543
305.2	ICD-9-CM	Nondependent cannabis abuse	6990
304.3	ICD-9-CM	Cannabis dependence	4832
T40.7	ICD-10-CM	Poisoning by, adverse effect of, and underdosing of cannabis (derivatives)	142
37344009	SNOMED CT	Cannabis abuse	136
428823006	SNOMED CT	Cannabis misuse	65
85005007	SNOMED CT	Cannabis dependence	53
191837001	SNOMED CT	Cannabis dependence, continuous	12

Diagnostic codes related to cannabis use are found in the dataset. Diagnostic codes with frequency <10 are not shown in the table.

In the ICD coding system, there is a hierarchical structure. By adding more characters to the code, the diagnosis becomes more specific. For example, for F12 in Table 6 the actual diagnosis codes were F12.20, F12.10, F12.90, etc. Here they are aggregated to the most general cases (e.g., parent codes) related to cannabis use.

### Reporting summary

Further information on research design is available in the Nature Research Reporting Summary linked to this article.

### Supplementary information

Supplementary Material

Reporting Summary

## Data Availability

The data used in this study cannot be publicly shared due to patient privacy concerns. Data can be accessed upon reasonable request pending approvals from Boston Children’s Hospital.

## Code availability

The main component in the proposed pipeline is the pre-trained BCH-BERT model, pretrained on clinical notes of Boston Children’s Hospital patients, which cannot be shared because of patient privacy concerns. The model and other scripts can be accessed upon reasonable request pending approvals from Boston Children’s Hospital.
