# Supplementary material for: Disparities in cannabis use and documentation in electronic health records among children and young adults
Source: NPJ Digit Med. 2023 Aug 8;6:138. doi: 10.1038/s41746-023-00885-w (PMC10409778; doi:10.1038/s41746-023-00885-w)
Supplement: Supplementary file 1 — Supplementary Material [file 41746_2023_885_MOESM1_ESM.pdf]

Supplementary Materials

Supplementary Table 1 Characteristics of patients with and without cannabis documentation excluding medical cannabis. P-values are calculated from generalized linear mixed models. aOR for female patients is calculated relative to the male patients. aOR for different races is calculated relative to white patients. The "Other" race group consists of Native American, Alaska native, native Hawaiian or other Pacific Islander, and multi-racial patients. For SVI, aOR is calculated per every 0.01 point change.

**Characteristics**	**Documented Patients**	**Not Documented Patients**	**aOR (95% CI)**	**P-Value**
Total Sample, No.	20,312	348,248	NR	NR
Sex, No (%)				
Female	10,739 (52.9%)	183,311(52.6%)	1.098 (1.061, 1.137)	**<.001**
Male	9,572 (47.1%)	164,901 (47.4%)	NR	NR
Unknown	1 (<0.1%)	36 (<0.1%)	NR	NR
Race, No. (%)				
Asian	365 (1.8%)	8,057 (2.3%)	0.826 (0.735, 0.928)	**.001**
Black	3,303 (16.3%)	14,403 (4.1%)	3.713 (3.514, 3.924)	**<.001**
Hispanic	3,427 (16.9%)	21,188 (6.1%)	2.362 (2.236, 2.495)	**<.001**
Other	1,134 (5.6%)	16,723 (4.8%)	1.200 (1.117, 1.290)	**<.001**
White	10,133 (49.9%)	195,849 (56.2%)	NR	NR
Unavailable	1,950 (9.6%)	92,028 (26.4%)	NR	NR
SVI, mean (SD)	0.308 (0.267)	0.269 (0.243)	1.001 (1.001, 1.002)	**<.001**

Dictionary of cannabis related keywords:

[marijuana, cbd, cannabis, thc, weed, mj, epidiolex, cannabidiol, marinol, dronabinol, syndros, cesamet, nabilone]

List of keywords considered as misspellings of marijuana:

[miajuana, mariujana, majiuana, marjiuana, marijuaan, marajuina, marijuanaa, marijuania, marijiuana, marijaua, marrijuanna, mirajauna, mirajuana, marjiuanna, marijauna, marijana, marijauana, majijuana, mariuanna, maraijuana, majauana, marajuan, marujauna, marijauan, marajuna, miarjuana, marijuanana, mairijuana, mairuana, marjjuana, marjuanna, marujuana, marijuahana, marijauaba, marajuauna, marajuanna, marinuana, mairjauna, mariajuana, marijauaa, marajiuana, majruana, marijuiana, marijuaa, marijauann, mjarijuana, marjuania, mairjuana, marjianua, maruijuanna, marinujana, marijanna, marujana, marjauna, marijaunua, mauirjuana, marijuauna, marjijuana, marajuia, marajana, majuana, mariujuana, mariuana, marijurana, marjuiana, marjuana, marajauna, marajuana, mariaja, marujanna, marijuanaat, marijunaa, marajanna, marijuaana, maijuana, marijuanna]

Filtering out notes related to common bile duct (CBD): if the clinical note is screened because of the keyword 'cbd' and any of the substrings in list CBD_KEY appear in it then it's flagged and filtered out. Note that the substrings in CBD_KEY can be anywhere in the clinical note, meaning words such as gastro, gastroenterology, gastrointestinal, etc. all match with gastro in CBD_KEY and cause the clinical note to be filtered out.

CBD_KEY = [abdominal, gallbladder, pancreas, common bile duct, cbd dilation, gastro]

Filtering out notes related to allergy detection (weed): if the clinical note is screened because of the keyword 'weed' and any of the substrings in list ALLERG_KEY appear in it then it's flagged and filtered out. Note that the substrings in ALLERG_KEY can be anywhere in the clinical note, meaning words such as allergy, allergic, allergens, etc. all match with allerg in ALLERG_KEY and cause the clinical note to be filtered out.

ALLERG_KEY = [pollen, allerg]
